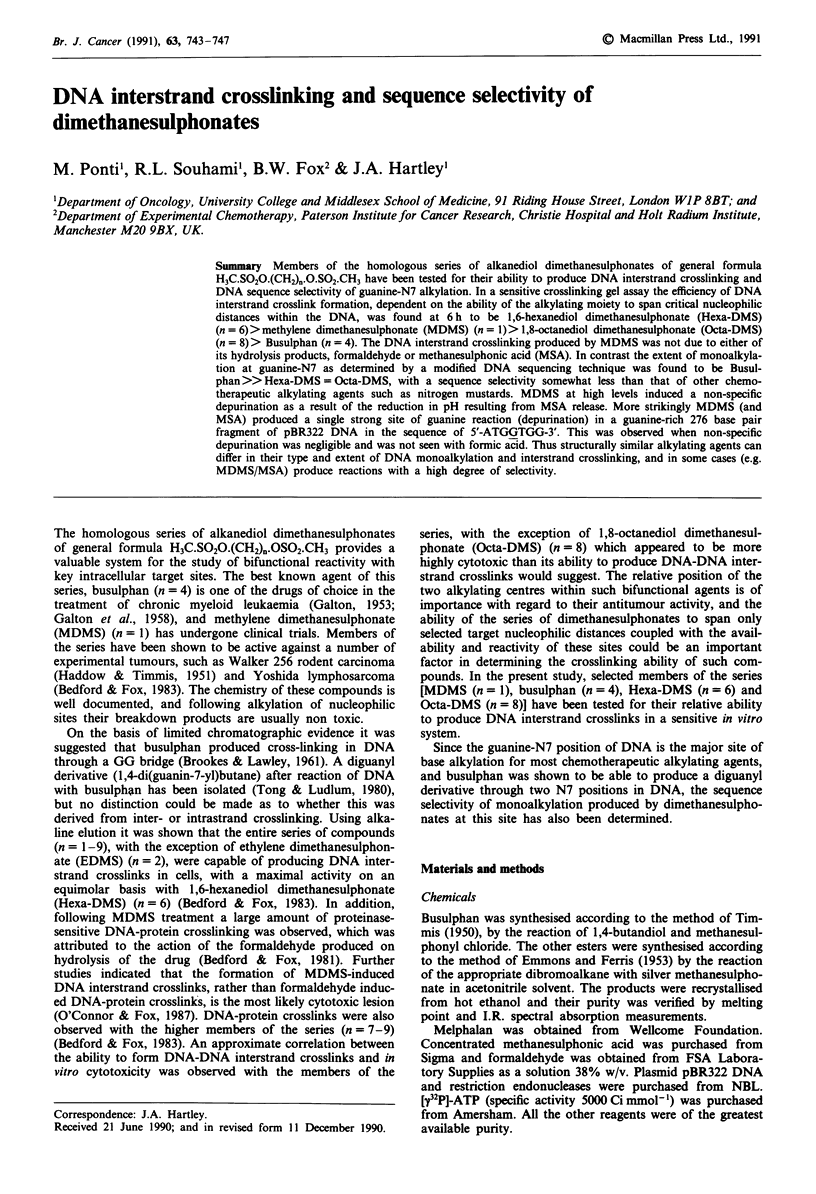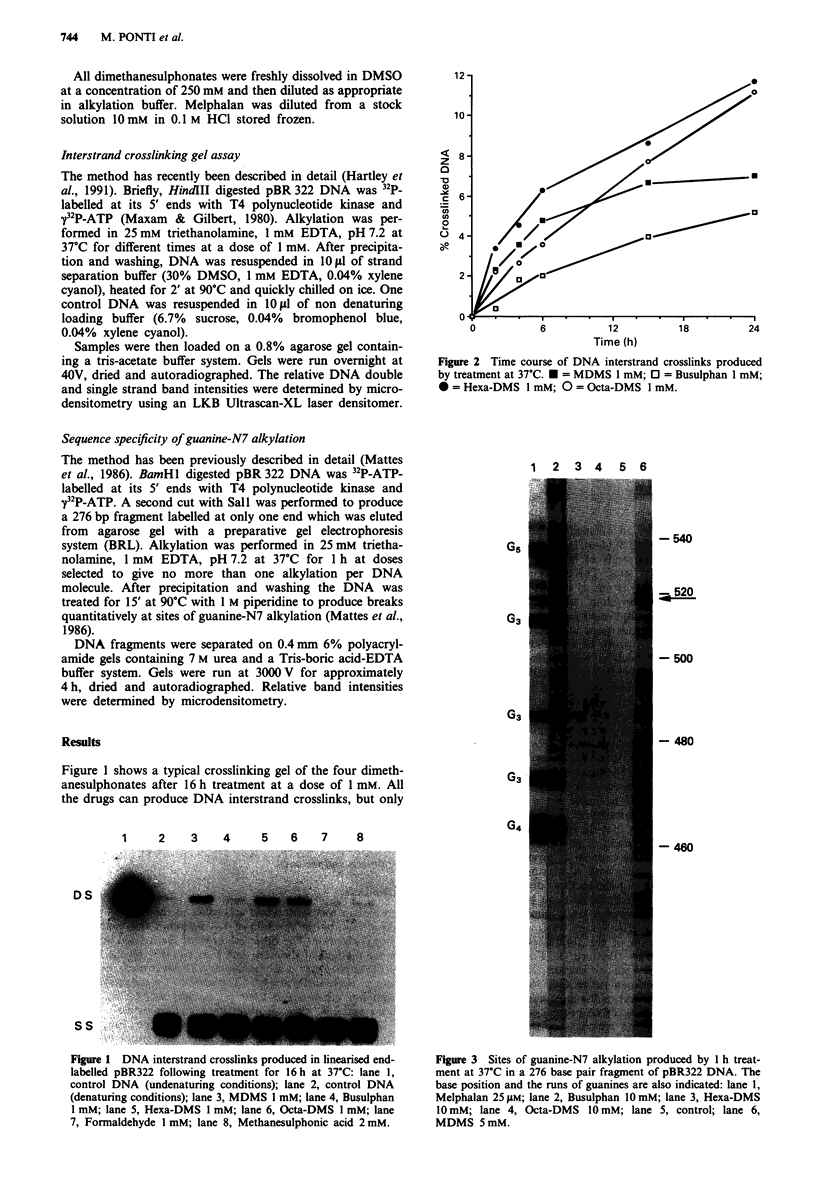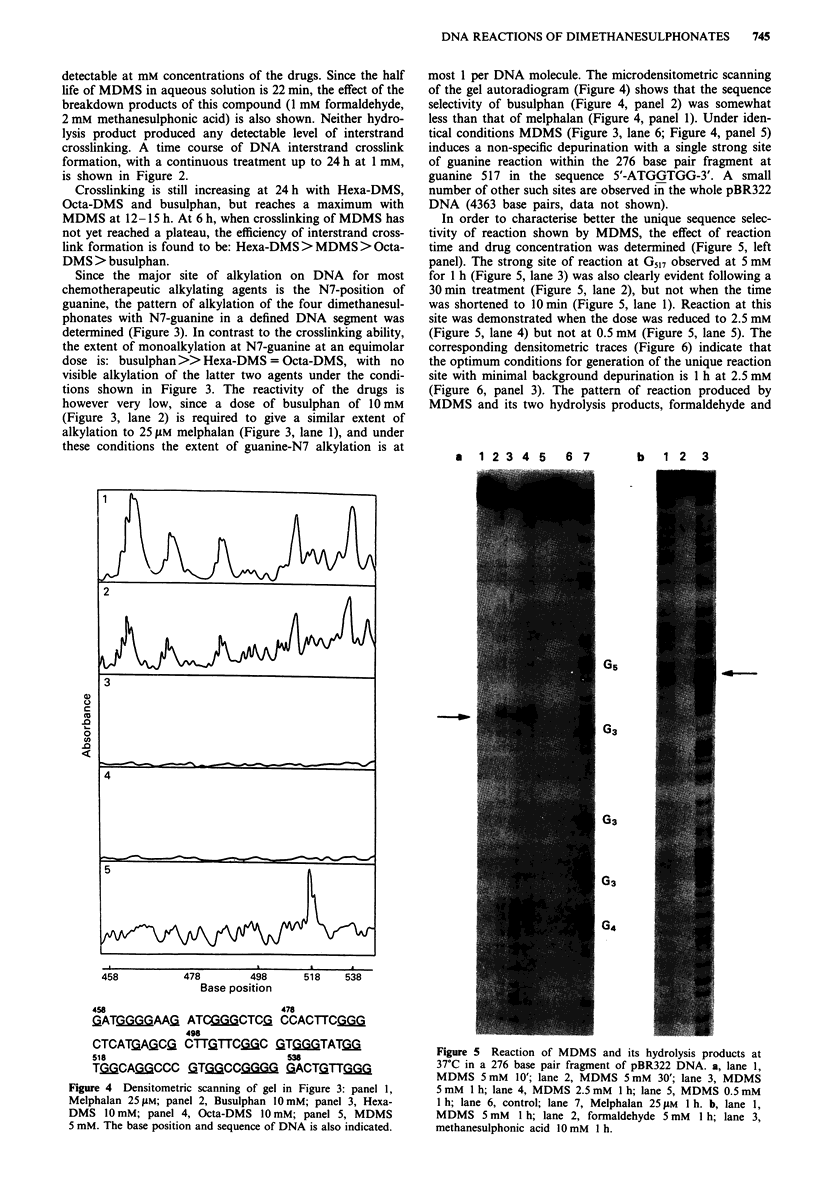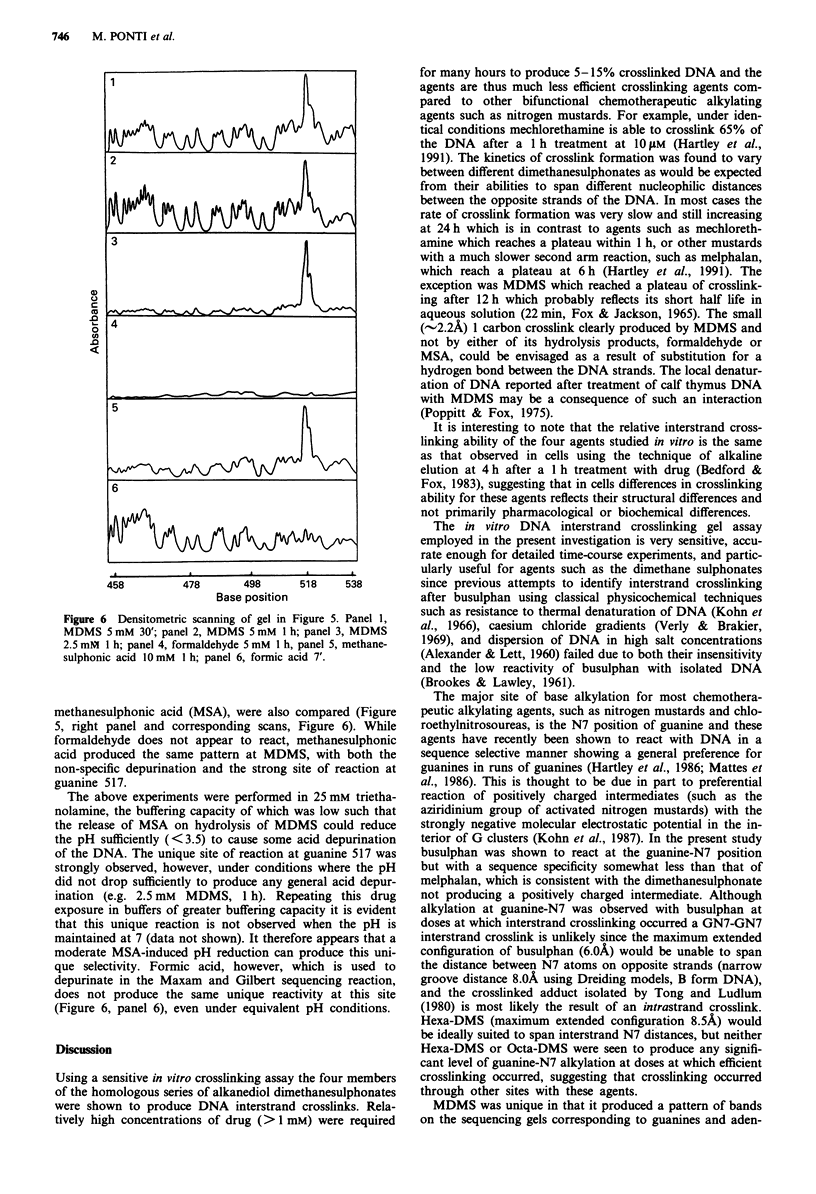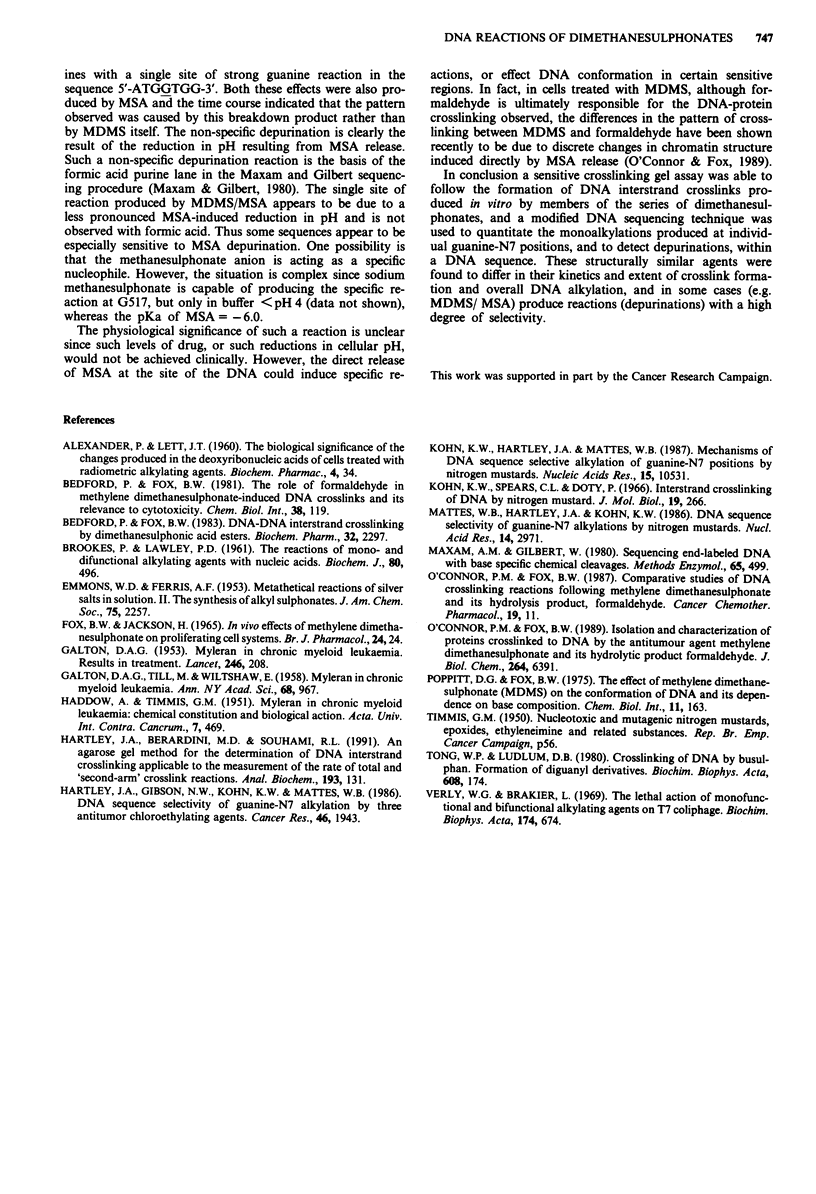# DNA interstrand crosslinking and sequence selectivity of dimethanesulphonates.

**DOI:** 10.1038/bjc.1991.166

**Published:** 1991-05

**Authors:** M. Ponti, R. L. Souhami, B. W. Fox, J. A. Hartley

**Affiliations:** Department of Oncology, University College and Middlesex School of Medicine, London, UK.

## Abstract

**Images:**


Br. J. Cancer (1991), 63, 743–747 © Macmillan Press Ltd., 1991

### DNA interstrand crosslinking and sequence selectivity of dimethanesulphonates

M. Ponti[1], R.L. Souhami[1], B.W. Fox[2] & J.A. Hartley[1]

[1]Department of Oncology, University College and Middlesex School of Medicine, 91 Riding House Street, London W1P 8BT; and [2]Department of Experimental Chemotherapy, Paterson Institute for Cancer Research, Christie Hospital and Holt Radium Institute, Manchester M20 9BX, UK.

**Summary** Members of the homologous series of alkanediol dimethanesulphonates of general formula $H_3C.SO_2O.(CH_2)_n.O.SO_2.CH_3$ have been tested for their ability to produce DNA interstrand crosslinking and DNA sequence selectivity of guanine-N7 alkylation. In a sensitive crosslinking gel assay the efficiency of DNA interstrand crosslink formation, dependent on the ability of the alkylating moiety to span critical nucleophilic distances within the DNA, was found at 6 h to be 1,6-hexanediol dimethanesulphonate (Hexa-DMS) ($n = 6$) > methylene dimethanesulphonate (MDMS) ($n = 1$) > 1,8-octanediol dimethanesulphonate (Octa-DMS) ($n = 8$) > Busulphan ($n = 4$). The DNA interstrand crosslinking produced by MDMS was not due to either of its hydrolysis products, formaldehyde or methanesulphonic acid (MSA). In contrast the extent of monoalkylation at guanine-N7 as determined by a modified DNA sequencing technique was found to be Busulphan >> Hexa-DMS = Octa-DMS, with a sequence selectivity somewhat less than that of other chemotherapeutic alkylating agents such as nitrogen mustards. MDMS at high levels induced a non-specific depurination as a result of the reduction in pH resulting from MSA release. More strikingly MDMS (and MSA) produced a single strong site of guanine reaction (depurination) in a guanine-rich 276 base pair fragment of pBR322 DNA in the sequence of 5'-ATGGTGG-3'. This was observed when non-specific depurination was negligible and was not seen with formic acid. Thus structurally similar alkylating agents can differ in their type and extent of DNA monoalkylation and interstrand crosslinking, and in some cases (e.g. MDMS/MSA) produce reactions with a high degree of selectivity.

The homologous series of alkanediol dimethanesulphonates of general formula $H_3C.SO_2O.(CH_2)_n.OSO_2.CH_3$ provides a valuable system for the study of bifunctional reactivity with key intracellular target sites. The best known agent of this series, busulphan ($n = 4$) is one of the drugs of choice in the treatment of chronic myeloid leukaemia (Galton, 1953; Galton *et al.*, 1958), and methylene dimethanesulphonate (MDMS) ($n = 1$) has undergone clinical trials. Members of the series have been shown to be active against a number of experimental tumours, such as Walker 256 rodent carcinoma (Haddow & Timmis, 1951) and Yoshida lymphosarcoma (Bedford & Fox, 1983). The chemistry of these compounds is well documented, and following alkylation of nucleophilic sites their breakdown products are usually non toxic.

On the basis of limited chromatographic evidence it was suggested that busulphan produced cross-linking in DNA through a GG bridge (Brookes & Lawley, 1961). A diguanyl derivative (1,4-di(guanin-7-yl)butane) after reaction of DNA with busulphan has been isolated (Tong & Ludlum, 1980), but no distinction could be made as to whether this was derived from inter- or intrastrand crosslinking. Using alkaline elution it was shown that the entire series of compounds ($n = 1-9$), with the exception of ethylene dimethanesulphonate (EDMS) ($n = 2$), were capable of producing DNA interstrand crosslinks in cells, with a maximal activity on an equimolar basis with 1,6-hexanediol dimethanesulphonate (Hexa-DMS) ($n = 6$) (Bedford & Fox, 1983). In addition, following MDMS treatment a large amount of proteinase-sensitive DNA-protein crosslinking was observed, which was attributed to the action of the formaldehyde produced on hydrolysis of the drug (Bedford & Fox, 1981). Further studies indicated that the formation of MDMS-induced DNA interstrand crosslinks, rather than formaldehyde induced DNA-protein crosslinks, is the most likely cytotoxic lesion (O'Connor & Fox, 1987). DNA-protein crosslinks were also observed with the higher members of the series ($n = 7-9$) (Bedford & Fox, 1983). An approximate correlation between the ability to form DNA-DNA interstrand crosslinks and *in vitro* cytotoxicity was observed with the members of the series, with the exception of 1,8-octanediol dimethanesulphonate (Octa-DMS) ($n = 8$) which appeared to be more highly cytotoxic than its ability to produce DNA-DNA interstrand crosslinks would suggest. The relative position of the two alkylating centres within such bifunctional agents is of importance with regard to their antitumour activity, and the ability of the series of dimethanesulphonates to span only selected target nucleophilic distances coupled with the availability and reactivity of these sites could be an important factor in determining the crosslinking ability of such compounds. In the present study, selected members of the series [MDMS ($n = 1$), busulphan ($n = 4$), Hexa-DMS ($n = 6$) and Octa-DMS ($n = 8$)] have been tested for their relative ability to produce DNA interstrand crosslinks in a sensitive *in vitro* system.

Since the guanine-N7 position of DNA is the major site of base alkylation for most chemotherapeutic alkylating agents, and busulphan was shown to be able to produce a diguanyl derivative through two N7 positions in DNA, the sequence selectivity of monoalkylation produced by dimethanesulphonates at this site has also been determined.

### Materials and methods

### Chemicals

Busulphan was synthesised according to the method of Timmis (1950), by the reaction of 1,4-butandiol and methanesulphonyl chloride. The other esters were synthesised according to the method of Emmons and Ferris (1953) by the reaction of the appropriate dibromoalkane with silver methanesulphonate in acetonitrile solvent. The products were recrystallised from hot ethanol and their purity was verified by melting point and I.R. spectral absorption measurements.

Melphalan was obtained from Wellcome Foundation. Concentrated methanesulphonic acid was purchased from Sigma and formaldehyde was obtained from FSA Laboratory Supplies as a solution 38% w/v. Plasmid pBR322 DNA and restriction endonucleases were purchased from NBL. [$\gamma^{32}$P]-ATP (specific activity 5000 Ci mmol$^{-1}$) was purchased from Amersham. All the other reagents were of the greatest available purity.

Correspondence: J.A. Hartley.
Received 21 June 1990; and in revised form 11 December 1990.

All dimethanesulphonates were freshly dissolved in DMSO at a concentration of 250 mM and then diluted as appropriate in alkylation buffer. Melphalan was diluted from a stock solution 10 mM in 0.1 M HCl stored frozen.

*Interstrand crosslinking gel assay*

The method has recently been described in detail (Hartley *et al.*, 1991). Briefly, *Hind*III digested pBR 322 DNA was $^{32}$P-labelled at its 5′ ends with T4 polynucleotide kinase and $\gamma^{32}$P-ATP (Maxam & Gilbert, 1980). Alkylation was performed in 25 mM triethanolamine, 1 mM EDTA, pH 7.2 at 37°C for different times at a dose of 1 mM. After precipitation and washing, DNA was resuspended in 10 µl of strand separation buffer (30% DMSO, 1 mM EDTA, 0.04% xylene cyanol), heated for 2′ at 90°C and quickly chilled on ice. One control DNA was resuspended in 10 µl of non denaturing loading buffer (6.7% sucrose, 0.04% bromophenol blue, 0.04% xylene cyanol).

Samples were then loaded on a 0.8% agarose gel containing a tris-acetate buffer system. Gels were run overnight at 40V, dried and autoradiographed. The relative DNA double and single strand band intensities were determined by microdensitometry using an LKB Ultrascan-XL laser densitomer.

*Sequence specificity of guanine-N7 alkylation*

The method has been previously described in detail (Mattes *et al.*, 1986). *Bam*H1 digested pBR 322 DNA was $^{32}$P-ATP-labelled at its 5′ ends with T4 polynucleotide kinase and $\gamma^{32}$P-ATP. A second cut with Sal1 was performed to produce a 276 bp fragment labelled at only one end which was eluted from agarose gel with a preparative gel electrophoresis system (BRL). Alkylation was performed in 25 mM triethanolamine, 1 mM EDTA, pH 7.2 at 37°C for 1 h at doses selected to give no more than one alkylation per DNA molecule. After precipitation and washing the DNA was treated for 15′ at 90°C with 1 M piperidine to produce breaks quantitatively at sites of guanine-N7 alkylation (Mattes *et al.*, 1986).

DNA fragments were separated on 0.4 mm 6% polyacrylamide gels containing 7 M urea and a Tris-boric acid-EDTA buffer system. Gels were run at 3000 V for approximately 4 h, dried and autoradiographed. Relative band intensities were determined by microdensitometry.

### Results

Figure 1 shows a typical crosslinking gel of the four dimethanesulphonates after 16 h treatment at a dose of 1 mM. All the drugs can produce DNA interstrand crosslinks, but only

**Figure 1** DNA interstrand crosslinks produced in linearised end-labelled pBR322 following treatment for 16 h at 37°C: lane 1, control DNA (undenaturing conditions); lane 2, control DNA (denaturing conditions); lane 3, MDMS 1 mM; lane 4, Busulphan 1 mM; lane 5, Hexa-DMS 1 mM; lane 6, Octa-DMS 1 mM; lane 7, Formaldehyde 1 mM; lane 8, Methanesulphonic acid 2 mM.

**Figure 2** Time course of DNA interstrand crosslinks produced by treatment at 37°C. ■ = MDMS 1 mM; □ = Busulphan 1 mM; ● = Hexa-DMS 1 mM; ○ = Octa-DMS 1 mM.

**Figure 3** Sites of guanine-N7 alkylation produced by 1 h treatment at 37°C in a 276 base pair fragment of pBR322 DNA. The base position and the runs of guanines are also indicated: lane 1, Melphalan 25 µM; lane 2, Busulphan 10 mM; lane 3, Hexa-DMS 10 mM; lane 4, Octa-DMS 10 mM; lane 5, control; lane 6, MDMS 5 mM.

detectable at mM concentrations of the drugs. Since the half life of MDMS in aqueous solution is 22 min, the effect of the breakdown products of this compound (1 mM formaldehyde, 2 mM methanesulphonic acid) is also shown. Neither hydrolysis product produced any detectable level of interstrand crosslinking. A time course of DNA interstrand crosslink formation, with a continuous treatment up to 24 h at 1 mM, is shown in Figure 2.

Crosslinking is still increasing at 24 h with Hexa-DMS, Octa-DMS and busulphan, but reaches a maximum with MDMS at 12–15 h. At 6 h, when crosslinking of MDMS has not yet reached a plateau, the efficiency of interstrand crosslink formation is found to be: Hexa-DMS > MDMS > Octa-DMS > busulphan.

Since the major site of alkylation on DNA for most chemotherapeutic alkylating agents is the N7-position of guanine, the pattern of alkylation of the four dimethanesulphonates with N7-guanine in a defined DNA segment was determined (Figure 3). In contrast to the crosslinking ability, the extent of monoalkylation at N7-guanine at an equimolar dose is: busulphan >> Hexa-DMS = Octa-DMS, with no visible alkylation of the latter two agents under the conditions shown in Figure 3. The reactivity of the drugs is however very low, since a dose of busulphan of 10 mM (Figure 3, lane 2) is required to give a similar extent of alkylation to 25 μM melphalan (Figure 3, lane 1), and under these conditions the extent of guanine-N7 alkylation is at most 1 per DNA molecule. The microdensitometric scanning of the gel autoradiogram (Figure 4) shows that the sequence selectivity of busulphan (Figure 4, panel 2) was somewhat less than that of melphalan (Figure 4, panel 1). Under identical conditions MDMS (Figure 3, lane 6; Figure 4, panel 5) induces a non-specific depurination with a single strong site of guanine reaction within the 276 base pair fragment at guanine 517 in the sequence 5′-ATGGTGG-3′. A small number of other such sites are observed in the whole pBR322 DNA (4363 base pairs, data not shown).

In order to characterise better the unique sequence selectivity of reaction shown by MDMS, the effect of reaction time and drug concentration was determined (Figure 5, left panel). The strong site of reaction at $G_{517}$ observed at 5 mM for 1 h (Figure 5, lane 3) was also clearly evident following a 30 min treatment (Figure 5, lane 2), but not when the time was shortened to 10 min (Figure 5, lane 1). Reaction at this site was demonstrated when the dose was reduced to 2.5 mM (Figure 5, lane 4) but not at 0.5 mM (Figure 5, lane 5). The corresponding densitometric traces (Figure 6) indicate that the optimum conditions for generation of the unique reaction site with minimal background depurination is 1 h at 2.5 mM (Figure 6, panel 3). The pattern of reaction produced by MDMS and its two hydrolysis products, formaldehyde and

458 ... 478

GATGGGGAAG ATCGGGCTCG CCACTTCGGG

498

CTCATGAGCG CTTGTTCGGC GTGGGTATGG

518 ... 538

TGGCAGGCCC GTGGCCGGGG GACTGTTGGG

**Figure 4** Densitometric scanning of gel in Figure 3: panel 1, Melphalan 25 μM; panel 2, Busulphan 10 mM; panel 3, Hexa-DMS 10 mM; panel 4, Octa-DMS 10 mM; panel 5, MDMS 5 mM. The base position and sequence of DNA is also indicated.

**Figure 5** Reaction of MDMS and its hydrolysis products at 37°C in a 276 base pair fragment of pBR322 DNA. **a**, lane 1, MDMS 5 mM 10′; lane 2, MDMS 5 mM 30′; lane 3, MDMS 5 mM 1 h; lane 4, MDMS 2.5 mM 1 h; lane 5, MDMS 0.5 mM 1 h; lane 6, control; lane 7, Melphalan 25 μM 1 h. **b**, lane 1, MDMS 5 mM 1 h; lane 2, formaldehyde 5 mM 1 h; lane 3, methanesulphonic acid 10 mM 1 h.

**Figure 6** Densitometric scanning of gel in Figure 5. Panel 1, MDMS 5 mM 30′; panel 2, MDMS 5 mM 1 h; panel 3, MDMS 2.5 mM 1 h; panel 4, formaldehyde 5 mM 1 h, panel 5, methanesulphonic acid 10 mM 1 h; panel 6, formic acid 7′.

methanesulphonic acid (MSA), were also compared (Figure 5, right panel and corresponding scans, Figure 6). While formaldehyde does not appear to react, methanesulphonic acid produced the same pattern at MDMS, with both the non-specific depurination and the strong site of reaction at guanine 517.

The above experiments were performed in 25 mM triethanolamine, the buffering capacity of which was low such that the release of MSA on hydrolysis of MDMS could reduce the pH sufficiently (<3.5) to cause some acid depurination of the DNA. The unique site of reaction at guanine 517 was strongly observed, however, under conditions where the pH did not drop sufficiently to produce any general acid depurination (e.g. 2.5 mM MDMS, 1 h). Repeating this drug exposure in buffers of greater buffering capacity it is evident that this unique reaction is not observed when the pH is maintained at 7 (data not shown). It therefore appears that a moderate MSA-induced pH reduction can produce this unique selectivity. Formic acid, however, which is used to depurinate in the Maxam and Gilbert sequencing reaction, does not produce the same unique reactivity at this site (Figure 6, panel 6), even under equivalent pH conditions.

### Discussion

Using a sensitive *in vitro* crosslinking assay the four members of the homologous series of alkanediol dimethanesulphonates were shown to produce DNA interstrand crosslinks. Relatively high concentrations of drug (>1 mM) were required for many hours to produce 5–15% crosslinked DNA and the agents are thus much less efficient crosslinking agents compared to other bifunctional chemotherapeutic alkylating agents such as nitrogen mustards. For example, under identical conditions mechlorethamine is able to crosslink 65% of the DNA after a 1 h treatment at 10 μM (Hartley *et al.*, 1991). The kinetics of crosslink formation was found to vary between different dimethanesulphonates as would be expected from their abilities to span different nucleophilic distances between the opposite strands of the DNA. In most cases the rate of crosslink formation was very slow and still increasing at 24 h which is in contrast to agents such as mechlorethamine which reaches a plateau within 1 h, or other mustards with a much slower second arm reaction, such as melphalan, which reach a plateau at 6 h (Hartley *et al.*, 1991). The exception was MDMS which reached a plateau of crosslinking after 12 h which probably reflects its short half life in aqueous solution (22 min, Fox & Jackson, 1965). The small (~2.2Å) 1 carbon crosslink clearly produced by MDMS and not by either of its hydrolysis products, formaldehyde or MSA, could be envisaged as a result of substitution for a hydrogen bond between the DNA strands. The local denaturation of DNA reported after treatment of calf thymus DNA with MDMS may be a consequence of such an interaction (Poppitt & Fox, 1975).

It is interesting to note that the relative interstrand crosslinking ability of the four agents studied *in vitro* is the same as that observed in cells using the technique of alkaline elution at 4 h after a 1 h treatment with drug (Bedford & Fox, 1983), suggesting that in cells differences in crosslinking ability for these agents reflects their structural differences and not primarily pharmacological or biochemical differences.

The *in vitro* DNA interstrand crosslinking gel assay employed in the present investigation is very sensitive, accurate enough for detailed time-course experiments, and particularly useful for agents such as the dimethane sulphonates since previous attempts to identify interstrand crosslinking after busulphan using classical physicochemical techniques such as resistance to thermal denaturation of DNA (Kohn *et al.*, 1966), caesium chloride gradients (Verly & Brakier, 1969), and dispersion of DNA in high salt concentrations (Alexander & Lett, 1960) failed due to both their insensitivity and the low reactivity of busulphan with isolated DNA (Brookes & Lawley, 1961).

The major site of base alkylation for most chemotherapeutic alkylating agents, such as nitrogen mustards and chloroethylnitrosoureas, is the N7 position of guanine and these agents have recently been shown to react with DNA in a sequence selective manner showing a general preference for guanines in runs of guanines (Hartley *et al.*, 1986; Mattes *et al.*, 1986). This is thought to be due in part to preferential reaction of positively charged intermediates (such as the aziridinium group of activated nitrogen mustards) with the strongly negative molecular electrostatic potential in the interior of G clusters (Kohn *et al.*, 1987). In the present study busulphan was shown to react at the guanine-N7 position but with a sequence specificity somewhat less than that of melphalan, which is consistent with the dimethanesulphonate not producing a positively charged intermediate. Although alkylation at guanine-N7 was observed with busulphan at doses at which interstrand crosslinking occurred a GN7-GN7 interstrand crosslink is unlikely since the maximum extended configuration of busulphan (6.0Å) would be unable to span the distance between N7 atoms on opposite strands (narrow groove distance 8.0Å using Dreiding models, B form DNA), and the crosslinked adduct isolated by Tong and Ludlum (1980) is most likely the result of an *intra*strand crosslink. Hexa-DMS (maximum extended configuration 8.5Å) would be ideally suited to span interstrand N7 distances, but neither Hexa-DMS or Octa-DMS were seen to produce any significant level of guanine-N7 alkylation at doses at which efficient crosslinking occurred, suggesting that crosslinking occurred through other sites with these agents.

MDMS was unique in that it produced a pattern of bands on the sequencing gels corresponding to guanines and adenines with a single site of strong guanine reaction in the sequence 5′-ATGGTGG-3′. Both these effects were also produced by MSA and the time course indicated that the pattern observed was caused by this breakdown product rather than by MDMS itself. The non-specific depurination is clearly the result of the reduction in pH resulting from MSA release. Such a non-specific depurination reaction is the basis of the formic acid purine lane in the Maxam and Gilbert sequencing procedure (Maxam & Gilbert, 1980). The single site of reaction produced by MDMS/MSA appears to be due to a less pronounced MSA-induced reduction in pH and is not observed with formic acid. Thus some sequences appear to be especially sensitive to MSA depurination. One possibility is that the methanesulphonate anion is acting as a specific nucleophile. However, the situation is complex since sodium methanesulphonate is capable of producing the specific reaction at G517, but only in buffer $<$pH 4 (data not shown), whereas the pKa of MSA = −6.0.

The physiological significance of such a reaction is unclear since such levels of drug, or such reductions in cellular pH, would not be achieved clinically. However, the direct release of MSA at the site of the DNA could induce specific reactions, or effect DNA conformation in certain sensitive regions. In fact, in cells treated with MDMS, although formaldehyde is ultimately responsible for the DNA-protein crosslinking observed, the differences in the pattern of crosslinking between MDMS and formaldehyde have been shown recently to be due to discrete changes in chromatin structure induced directly by MSA release (O'Connor & Fox, 1989).

In conclusion a sensitive crosslinking gel assay was able to follow the formation of DNA interstrand crosslinks produced *in vitro* by members of the series of dimethanesulphonates, and a modified DNA sequencing technique was used to quantitate the monoalkylations produced at individual guanine-N7 positions, and to detect depurinations, within a DNA sequence. These structurally similar agents were found to differ in their kinetics and extent of crosslink formation and overall DNA alkylation, and in some cases (e.g. MDMS/ MSA) produce reactions (depurinations) with a high degree of selectivity.

This work was supported in part by the Cancer Research Campaign.

### References

ALEXANDER, P. & LETT, J.T. (1960). The biological significance of the changes produced in the deoxyribonucleic acids of cells treated with radiometric alkylating agents. *Biochem. Pharmac.*, **4**, 34.

BEDFORD, P. & FOX, B.W. (1981). The role of formaldehyde in methylene dimethanesulphonate-induced DNA crosslinks and its relevance to cytotoxicity. *Chem. Biol. Int.*, **38**, 119.

BEDFORD, P. & FOX, B.W. (1983). DNA-DNA interstrand crosslinking by dimethanesulphonic acid esters. *Biochem. Pharm.*, **32**, 2297.

BROOKES, P. & LAWLEY, P.D. (1961). The reactions of mono- and difunctional alkylating agents with nucleic acids. *Biochem. J.*, **80**, 496.

EMMONS, W.D. & FERRIS, A.F. (1953). Metathetical reactions of silver salts in solution. II. The synthesis of alkyl sulphonates. *J. Am. Chem. Soc.*, **75**, 2257.

FOX, B.W. & JACKSON, H. (1965). *In vivo* effects of methylene dimethanesulphonate on proliferating cell systems. *Br. J. Pharmacol.*, **24**, 24.

GALTON, D.A.G. (1953). Myleran in chronic myeloid leukaemia. Results in treatment. *Lancet*, **246**, 208.

GALTON, D.A.G., TILL, M. & WILTSHAW, E. (1958). Myleran in chronic myeloid leukaemia. *Ann. NY Acad. Sci.*, **68**, 967.

HADDOW, A. & TIMMIS, G.M. (1951). Myleran in chronic myeloid leukaemia: chemical constitution and biological action. *Acta. Univ. Int. Contra. Cancrum.*, **7**, 469.

HARTLEY, J.A., BERARDINI, M.D. & SOUHAMI, R.L. (1991). An agarose gel method for the determination of DNA interstrand crosslinking applicable to the measurement of the rate of total and 'second-arm' crosslink reactions. *Anal. Biochem.*, **193**, 131.

HARTLEY, J.A., GIBSON, N.W., KOHN, K.W. & MATTES, W.B. (1986). DNA sequence selectivity of guanine-N7 alkylation by three antitumor chloroethylating agents. *Cancer Res.*, **46**, 1943.

KOHN, K.W., HARTLEY, J.A. & MATTES, W.B. (1987). Mechanisms of DNA sequence selective alkylation of guanine-N7 positions by nitrogen mustards. *Nucleic Acids Res.*, **15**, 10531.

KOHN, K.W., SPEARS, C.L. & DOTY, P. (1966). Interstrand crosslinking of DNA by nitrogen mustard. *J. Mol. Biol.*, **19**, 266.

MATTES, W.B., HARTLEY, J.A. & KOHN, K.W. (1986). DNA sequence selectivity of guanine-N7 alkylations by nitrogen mustards. *Nucl. Acid Res.*, **14**, 2971.

MAXAM, A.M. & GILBERT, W. (1980). Sequencing end-labeled DNA with base specific chemical cleavages. *Methods Enzymol.*, **65**, 499.

O'CONNOR, P.M. & FOX, B.W. (1987). Comparative studies of DNA crosslinking reactions following methylene dimethanesulphonate and its hydrolysis product, formaldehyde. *Cancer Chemother. Pharmacol.*, **19**, 11.

O'CONNOR, P.M. & FOX, B.W. (1989). Isolation and characterization of proteins crosslinked to DNA by the antitumour agent methylene dimethanesulphonate and its hydrolytic product formaldehyde. *J. Biol. Chem.*, **264**, 6391.

POPPITT, D.G. & FOX, B.W. (1975). The effect of methylene dimethanesulphonate (MDMS) on the conformation of DNA and its dependence on base composition. *Chem. Biol. Int.*, **11**, 163.

TIMMIS, G.M. (1950). Nucleotoxic and mutagenic nitrogen mustards, epoxides, ethyleneimine and related substances. *Rep. Br. Emp. Cancer Campaign*, p56.

TONG, W.P. & LUDLUM, D.B. (1980). Crosslinking of DNA by busulphan. Formation of diguanyl derivatives. *Biochim. Biophys. Acta*, **608**, 174.

VERLY, W.G. & BRAKIER, L. (1969). The lethal action of monofunctional and bifunctional alkylating agents on T7 coliphage. *Biochim. Biophys. Acta*, **174**, 674.